# Intention of Collaboration among Dental Students during the COVID-19 Pandemic

**DOI:** 10.3390/dj10030040

**Published:** 2022-03-08

**Authors:** Kyriaki Hadjichambi, Evie Georgiadou, Vasileios Margaritis, Maria Antoniadou

**Affiliations:** 1 Dental School, National and Kapodistrian University of Athens, 11527 Athens, Greece; hadjichambik@gmail.com (K.H.); eviegeox@gmail.com (E.G.); 2 Senior Public Health Faculty, College of Health Professions, Walden University, Minneapolis, MN 55401, USA; vasileios.margaritis@mail.waldenu.edu

**Keywords:** collaboration, COVID-19, dental education, dentistry, motivation, teamwork

## Abstract

Interpersonal communication skills (ICS) are crucial for effective dental practice and interprofessional collaboration. The current study aimed to assess the attitudes of Greek dental undergraduate students towards team working and their cooperation abilities during the COVID-19 pandemic. One-hundred and twenty-seven fourth-semester dental students (N_1_ = 127) out of 145 (N_0_) filled in the online survey placed on Google forms. The “Dental Students Cooperation Questionnaire” (DSC) consisted of 49 questions and was available for completion for one week during April 2020. Bivariate (ANOVA) and linear regression analysis of data revealed that mean scores of the questionnaire increased as the parents’ educational level also increased. Data analysis showed that dental students had the required ICS and the intention to collaborate with each other. Many participants managed to achieve group goals, were willing to support other members to fulfill the project’s goals, and there was no competition among them. They acknowledged the importance of feedback, the reward at the end of a group project and social media as a tool for teamworking communication. The students reported that the most important characteristics of an academic teacher were patience, willingness to cooperate, friendliness, politeness, willingness to help, accessibility and availability. It is suggested that group work should be included in the curriculum of dental schools to enhance the integration and evolution of students’ ICS, and the DSC questionnaire can be an effective tool to assess these skills.

## 1. Introduction

Collaboration and communication are two of the most crucial soft skills dentists should have to better understand patients’ and colleagues’ needs and attitudes [1,2]. Although recent data reveal that in terms of health units’ management, coordination and cooperation on team working can bring practical and economic benefits to the organization [3,4], it is important to recognize that this issue has not yet been adequately investigated in the field of dentistry. Before the blast of the COVID-19 pandemic, based on a learner-centered pedagogical method called team-based learning (TBL), it was noted that working in groups could allow dental students to apply more easily what they have learned while making treatment decisions [5]. Further, research has revealed the need for dental students to acquire more effective communication skills when collaborating in teams with others and applying knowledge in new dental cases [6]. Furthermore, teamwork could lead dental students to adopt a learning approach while they seek deeper meaning and personal relevance to each subject they learn [7]. Overall, collaboration and development of dental students’ communication skills could lead them to better connect what they have learned in theory to clinical practice [8].

As it is described so far, interpersonal communication and collaboration skills (ICS) include having respect, being attentive, showing compassion to nonverbal contact, being present, showing concern and interest in the patient and being aware of the importance of any kind of relationship and attitude corresponding to empathy [9,10]. Empathy is related to satisfaction, patient compliance, acceptance of the service [11] and better clinical outcomes [12]. In this sense, ICS are a group of personality traits, language, personal habits, social graces, communication, optimism and friendliness that affect relationships with others [13]. Besides this, it is known that efficient ICS of dental students improve their diagnostic performance, while they achieve better clinical and ethical decision making [14]. Finally, it seems that students themselves believe that ICS are important for a successful dental practice [15].

Within this context, the Council of European Dentists in their CED resolution suggested that ICS along with technical skills are equally important in developing good dental practice management. They discussed a future dentist who is: (a) a communicator, meaning he has a good knowledge of communication and (b) a collaborator, meaning he has knowledge and training on interprofessional and intraprofessional collaboration [16]. The American Dental Education Association also included ICS in the list of main capabilities expected for graduates from general dental education programs [17].

For two years now, humanity has been experiencing a public health crisis, the COVID-19 pandemic, that has already led to more than 246 million cases and approximately 5 million deaths worldwide [7]. With the global rapid increase in COVID-19 cases, leading to lockdowns and social-distancing, there was an imperative need for dental academic institutions to offer e-learning modules that could support part of the learning process otherwise exercised by physical presence [17,18,19,20,21]. Intraprofessional and interprofessional collaboration and teamwork through online modules were often imperative to facilitate and support the learning process and the exam system worldwide [22,23,24,25]. In this way, the learning process of technical skills and theoretical knowledge of dental subjects and the cultivation of ICS were accomplished at the same time by teamwork assignments [26].

Even though collaborative training forced by the pandemic was supposed to offer a positive professional identity, improve communication level and students’ knowledge and appreciation of other relevant professions, and enhance the mutual respect and acceptance among partners [5,6,7], there are no studies about the effect of teamwork in improving the collaborating ability of dental students, especially during a health crisis such as the pandemic. For this purpose, in this study, we aimed to evaluate the attitudes of dental students towards team working during COVID-19 restrictions. Further, we investigated aspects of interpersonal communication skills required for effective dental team collaboration. Lastly, we reported the basic characteristics that an academic teacher should have to better interact with dental students during collaborative teamworking.

## 2. Materials and Methods

For this study, all fourth-semester dental students on the undergraduate program of studies (N_0_ = 145), at the dental school of Athens, Greece, (academic year 2019–2020) were invited to fill in an online survey placed on Google forms, concerning aspects of a planned group project of the Operative Dentistry Laboratory course (Operative Dentistry I). The group project was assigned on a voluntary base within the period of the first lockdown due to the COVID-19 pandemic to cover part of the learning process of the subject for the period of the relevant academic semester. The themes were applied in the e-class of the subject and teams could choose their own among the list. There was a grade reward for the participants in the group project. This group assignment was their first effort to enroll students in team working since the beginning of their studies, according to the program curriculum, as designed before the pandemic.

The questionnaire was approved by the ethical committee of the department of Operative Dentistry, Dental School of Athens (no. A3, 28 March 2020). The link to the Google forms, consent form and questionnaire was emailed to each one of the participants on 10 April 2020 and asked to be filled in within a period of one week. Every student had the same chance to participate and fill in the questionnaire by him/herself. Details about the completion of the questionnaire were described in the introduction of the online form. No personal data or other identifiers were collected by completing the form and anonymous submission was guaranteed by the program. The message to complete the survey was sent twice to all participants, within a period of one week. No late responses were collected after this period and the questionnaire was closed to new answers. Furthermore, there was no compensation of any kind for participating in the study. Data were then collected and analyzed by the same researcher.

A mixed method design was followed for the Dental Students Cooperation questionnaire (DSC) used in the study, including both closed-ended and open-ended questions. Mixed research methods are widely used in health sciences. As already reported, they can contribute to a more comprehensive and in-depth picture than a standalone quantitative or qualitative study, as they integrate benefits of both methods [22]. The questions in DSC were arranged using a five-point Likert rating (1 Strongly disagree, 2 Disagree, 3 Neutral, 4 Agree and 5 Strongly agree). Likert-scale questionnaires are the most used type of instrument for measuring variables, given that they allow researchers to gather large amounts of data with relative ease [27].

**Figure 1:**
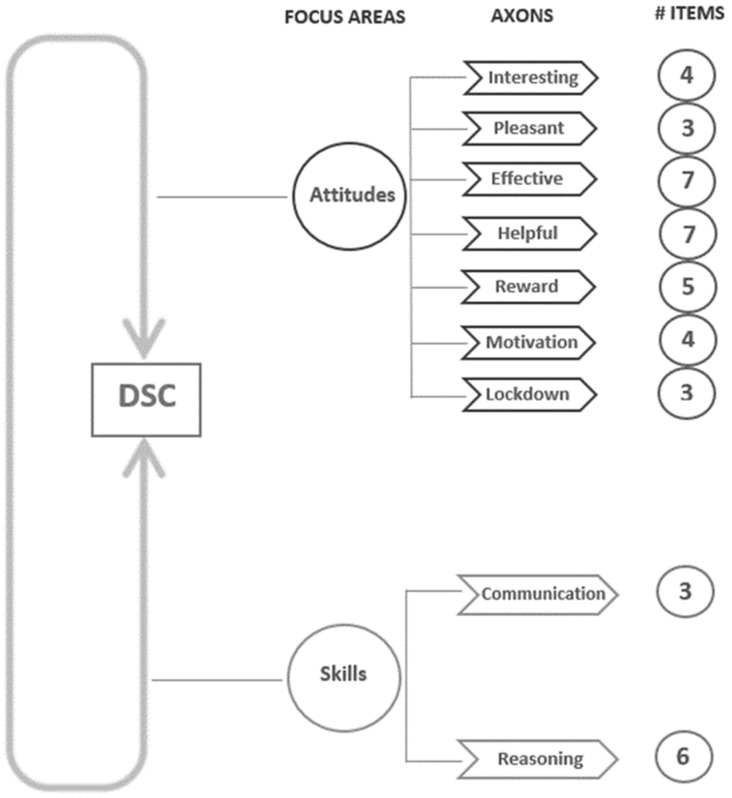
Research parts of the DSC questionnaire.

The DSC questionnaire consists of four parts. The first three parts include quantitative questions, while the fourth part consists of one quantitative and one open-ended qualitative question (Figure 1).

The questionnaire has been developed by taking elements from relevant studies of the field addressing issues on attitudes (effects of reward, influence of students’/parents’ educational level, motivation [6,15,18] and skills (cooperation and communication skills) [28,29,30,31,32,33] and placed in the section of Google forms (Appendix A).

The first part of the questionnaire included the demographic characteristics of the population under study (gender of the students, their parents’ educational level, their previous participation in teamwork and their anticipated workplace). The second part (B1) was focused on the area of students’ attitudes towards communication and cooperation during the project. The focus area included seven axons, investigating whether the students found the group project interesting, pleasant, effective and helpful as well as their motivation. The third part (C1 and C2) was focused on students’ communication and reasoning skills as an outcome of the group project. The fourth part’s items were more specific about the reward and the type of academic teacher they would prefer while working on team assignments. Some questions were based in previous studies (Q4, Q9, Q10, Q14 on the study [6], Q20, Q21 on [18], Q8, Q15, Q16 were modified from [28], Q24 on [29], Q25 on [30] and finally Q30, Q31, Q32, Q33 on [30,31,32,33], respectively. All other questions were specifically designed for the DSC questionnaire by the authors.

The construct validity of this survey instrument was confirmed by a panel of three experts on dental education, professors from the Dental School of Athens, who reviewed and revised the survey questions to be relevant to the topic. In addition, the instrument was pilot tested on 10 students to confirm its comprehensibility and usability.

For each closed-ended Likert scale question, mean scores were calculated by gender, parents’ educational level and anticipated place of dental practice of the students. The mean scores (outcome variables) were normally distributed, thus parametric tests (t-test for gender and ANOVA for parents’ educational level and place of dental practice) were conducted to explore potential associations between gender, parents’ educational level and anticipated place of dental practice and mean scores of each survey question. In addition, linear regression models were applied including different combinations of the independent variables used in the bivariate analyses above. All reported probability values (*p* values) were compared with a significance level of 5% (*p* < 0.05). The analysis of coded data was performed using the IBM SPSS Statistics for Windows, Version 27.0 Armonk, NY: IBM Corp (Released 2020). Qualitative content analysis was also carried out to analyze students’ responses to the open-ended question. This provided direct insights into the dental students’ thoughts about the type of academic teacher they would prefer.

## 3. Results

One hundred and twenty-one students (N_1_ = 127), out of 145 (N_0_) students, voluntarily completed the online questionnaire (response rate 87.6%). The survey instrument demonstrated satisfactory reliability (Cronbach’s alpha = 0.93). From the 127 participants, 48 (37.8%) were male and 79 (62.2%) females. The parental educational level was, elementary/junior high 4 (3.1%), high school 19 (15%) and university 104 (81.9%). The anticipated place of their future dental practice was, in a city 79 (62.2%), in a town/village 9 (7.1%), on an island 8 (6.3%), abroad 22 (17.3%) and no clinical practice was the option for 9 students (7.1%). The total scores for each part of the questionnaire by gender, parents’ educational level and future workplace of students as well the inferential analyses are presented in Table 1.

The students had a high score (>3) in almost all survey questions (data not shown), reflecting a high level of communication, cooperation and reasoning attitudes and skills. Especially the questions “We communicated through social media during lockdown to finish the project”, “We managed to achieve group goals” and “All the members contributed equally” received the highest score by the students (4.16, 4.10, 4.06, respectively). Furthermore, the students did not agree that “The competition among the members caused conflicts, which negatively affected the goal of the project” and that “There were differences between the group members” (1.46 and 2.01 mean score, respectively). The bivariate analysis of the data (ANOVA) revealed that the mean scores of the questionnaire increased as the parents’ educational level also increased (F = 3.374, *p* < 0.037). This predictor was the only one which remained significant after applying linear regression models (b = 0.227, *p* < 0.04). Linear regression also yielded an effect size of f^2^ = 0.15, which was used for performing post hoc power analysis using the G* Power calculator [34]. The achieved power was very satisfactory (0.93) confirming the adequacy of the sample size.

In addition, after combining and grouping students’ responses to the open-ended questions about the reward and the type of academic teacher they would prefer, it was found that the most frequently reported incentive was a better term grade (70.9%) (Table 2), while a patient and cooperative personality are the most desired characteristics of an academic teacher (22%) in coaching a teamwork project (Table 3).

## 4. Discussion

The World Health Organization (WHO) refers to the interprofessional collaboration among students, stating that working in groups is a key step in the integration and strengthening of health systems [35]. Furthermore, it is reported that organizational culture and expectations have a strong impact on health professionals’ participation and experience of teamwork education [4].

In studies particularly examining dental students’ ICS required for successful collaboration, the effective healthcare professional communication with patients (active listening and doctor’s empathy) is supposed to affect most general health outcomes and overall patient experience [36,37,38,39]. In addition to this, new pedagogical learning methods such as the one called cooperative learning (CL), helps to develop ICS in problem solving and critical thinking, primarily for students sharing ideas in team working [34]. Thus, effective communication strengthens teamworking and then collaboration offers the opportunity of critical thinking and quality assurance in dental services [40,41,42].

However, collaborating in group projects is not always easy as equity of contribution and fair reward seems problematic, while some participants may also be unwilling to pursue group goals [43] or not equally contributing to the task [6]. In our study though, many dental students managed to achieve group goals (Q9) and most members contributed equally to team working (Q10). Furthermore, many participants were willing to support the rest of the members to achieve the project’s goals (Q11), by developing a sense of community. In addition, studies suggest that students who cooperate better manage to deal more efficiently with problems that arise during the task [44]. Therefore, working in groups can create a cooperative community that helps each member to perform better [42]. On the other hand, literature supports that negative interdependence between team members often leads to competitive behavior and so individuals may discourage each other’s attempt to succeed [45]. In our case, most of the dental students believed that there was no competition among group members and therefore there was no negative impact on the goal of the project from such a behavior (Q14).

Another interesting issue is the importance of feedback throughout the project as well as intermediate evaluation of the students, which a remarkable number of participants considered as crucial [(Q27) “It would motivate me to work harder if we were examined more often and not just at the end of the work”]. This is also found in similar work since it is reported that not only timing but also type of feedback has an impact on students’ motivation for participating in extra-credit assignments [46]. In this sense, more regular feedback could motivate students to perform better [47].

It is a fact that COVID-19 not only engaged universities and schools all over the world to use e-learning as the main instruction method, but it also encouraged educators to use it as a vital method to build up students’ motivation and educational level [48]. In the same way, it has been demonstrated that students are more likely to be motivated by using e-learning and modern technology [49]. Furthermore, several studies suggest that students have positive perceptions about virtual hospitals, preclinical phantom courses and mobile applications and as a result e-learning could increase teaching and learning methods in clinical programs [22,48,49,50]. In our study, through the DSC questionnaire, social media was an important learning tool for dental students not only for communication but also for collaboration reasons by accomplishing the teamwork easier and faster [(Q31) “We communicated through social media during lockdown to finish the project”].

Another important part of the present study was students’ perception about a reward at the end of the group project as incentive to participate [(Q23) “A reward at the end of a group project would push me to participate in the project that I would not normally be interested in]”. On this point, it is supportive to our study that neuroscientists determined the circuitry of reward in the brain and have found that reward has the following three different mechanisms: liking, wanting, learning [51]. More specifically, neuroscience research suggests that reward improves attention, impacts behavior, enhances memory and decreases reaction times. Regarding the incentive for optimal cooperation, students in our study seemed to be willing to collaborate, especially for receiving better term grades and a recommendation letter or participating in a future project. However, compensation and a certificate of participation were less favored by study participants. Previous studies agree that grades and getting students’ attention are both rated as motivators and preferred rewards [52]. In general, reward and motivation are noted as factors that contribute to better team outcomes. It is reported that structure and distribution of rewards have an impact on the motivation of every single team member, affect the coordination and interdependence within teams and increase the quality of the team process that develops among group members [53,54,55]. As also discussed in other studies, students’ motivation is a criterion of academic performance and it is fundamental for effective education [56,57]. Thus, introducing a grade motivation system might enhance dental students’ interest in participating and working in teams. Of course, in this case, extrinsically motivated students have poor performance in contrast to intrinsically motivated students, who perform better academically for their own interest and learning [58,59,60]. Despite this limitation, motivation through some sort of reward, may act as a self-fulfilling prophecy, so students who set higher goals are more willing to make the extra effort required and end up achieving it [61,62,63]. This was also demonstrated in our study.

Another serious aspect that was also examined here, was the characteristics of the academic staff that seem to impact and motivate students’ teamwork (QD2). According to the study’s results, patience is among the most expected characteristics of an academic teacher responsible for teamwork. Students also want him/her to be cooperative, friendly, polite as well as accessible and available when students are in need. So, if the educator is supportive and can develop students’ interest in learning as well as their self-confidence, this could lead to a more dynamic learning process and better academic achievement [62,63]. As also mentioned elsewhere, health students highly value teamwork education programs that are implemented by facilitators who create practical authentic learning opportunities and foster reflection and debriefing for participants [4]. Similarly, it was found elsewhere that instructors who promoted understanding could better manage the classroom, while their enthusiasm could be the most important factor for students’ motivation [64,65,66]. It is already stated that in cooperative learning there is a positive interaction among students and a helpful instructor [67,68]. Overall, instructors and parents are the most important mediators in the development of student’s motivation in teamwork [62,66,67]. While parents help their children develop their attitude towards life and learning, the educator seems to have a stronger impact on students’ behavior especially about learning [68,69].

Furthermore, parents’ educational level and socioeconomic status have been found to have an important role as predictors of children’s academic success [67]. In our study too, the only statistically significant result was parents’ socioeconomic and educational status, as a precursor to their children’s collaboration skills. This study showed that the higher the parents’ education level was, the more collaborative the student. This is probably because these two factors, the socioeconomic and educational level, enable parents to acquire and model problem-solving strategies and social skills conducive to students’ academic success as also mentioned elsewhere [69,70,71]. It is further reported that a higher parental education level corresponds to a development of intelligence theory that motivates people to explore knowledge and human interaction without negative emotions of failure or fear. It is supposed to be even better if both parents have the same intelligence theory to pass on this behavior to their children. However, even if this is not the case within a couple, the mother’s role in education seems to be the most significant [72]. Generally, students that were treated as capable and intelligent by their mother or both their parents and teachers, grew up to be more powerful and were motivated to succeed and collaborate [70].

Overall, ICS required for effective team collaboration are conflict management, active listening, critical thinking, decision making, problem-solving, trust and self-awareness [71]. Students’ responses in this study confirmed that Greek dental students had the required ICS and the efficiency to work and collaborate during the pandemic. It is a serious finding that the educational level of their parents significantly affects their attitude towards collaboration, thus suggesting that overall high educational level can offer successful team working in dental health settings. Group working in dental education should be investigated further to improve academic performance, excellency, cooperation and communication skills among new dental professionals. This is a significant conclusion, as empowerment of the dental team through common goals is expected to improve sustainability in dental settings and quality of dental services [72].

## 5. Limitations of the Study

The questionnaire was given only to dental students of the fourth semester of the undergraduate program. Future approaches should enroll students from different semesters, with different dental training levels and disciplines. In addition, there should also be a future investigation of the responses of both undergraduate and postgraduate students since they use different cooperative learning processes and structures. It would also be important to report on whether the same students participating in the study during the pandemic could change behavior in non-pandemic learning circumstances or when the team assignment would be obligatory or the theme not interesting enough. The next research approach should further search whether a fidelity simulation used with specific communication strategies provides a powerful learning opportunity for students to practice teamwork skills and whether they have increased confidence after teamworking to be motivated to apply their newly learnt teamwork skills into their daily practice. Finally, it would be interesting to have insights on how successful dental students’ teams’ function to develop successful teamwork education programs and enhance the experience of participants.

## 6. Strengths of the Study

The present study addresses for the first time the issue of collaboration as a soft skill in forming productive and successful new dental professionals during a stressful period such as the pandemic restrictions and lockdowns. It also offers insights about the academic profile and the reward system needed for a teamworking learning approach in dental curriculums. The DSC questionnaire of the study is a newly designed and effective tool for addressing further collaborative issues in the dental education field and can offer the base for future investigation in the field.

## 7. Conclusions

Data from this study suggested that most dental students aim to work together to support the group’s common goal without any competition involved. Undergraduate dental students appear to have the required ICS and the efficiency to work together and collaborate. The socioeconomic and educational level of parents is significant in determining the degree of students’ collaborator profile. The higher the socioeconomic and educational level of parents, the more collaborative are the students. Furthermore, feedback, reward and social media are highlighted in this study as important factors for effective collaboration. Overall, dental students want their supervisor to be “patient, cooperative”, “friendly, polite” and “willing, accessible, available” to work together efficiently on an assigned project. The DSC questionnaire proved to be an effective measuring tool for collaborator’s skills in the dental field.

## Figures and Tables

**Table 1:** Bivariate (ANOVA) and linear regression analysis of the total mean scores (M) and standard deviations (SD) of the DSC questionnaire by gender, parent’s educational level and anticipated place of practice of students.

	Gender				Parents’ Educational Level *						Anticipated Place of Dental Practice											
	Male		Female		Elementary Junior High		High School		University		City		Town Village		Island		Abroad		No Clinical Practice		Total	
	M	SD	M	SD	M	SD	M	SD	M	SD	M	SD	M	SD	M	SD	M	SD	M	SD	M	SD
B1 survey	3.21	0.50	3.10	0.62	2.47	1.68	3.28	0.47	3.15	0.51	3.15	0.61	3.27	0.39	3.03	0.57	3.21	0.47	2.87	0.65	3.14	0.58
C1–C2 Survey	3.2	1.2	3.0	1.0	2.3	1.9	3.2	1.2	3.1	1.0	3.1	1.1	3.3	0.6	2.7	0.7	3.3	1.2	2.5	1.2	3.1	1.1

* ANOVA: F = 3.374, *p* < 0.037; Linear regression: b = 0.227, *p* < 0.04.

**Table 2:** Incentive for optimal cooperation.

Incentive for Optimal Cooperation	Ν	%
Receive better term grade	90	70.9
Compensation	8	6.3
Certificate of participation	11	8.7
Other (e.g., participation in future projects, recommendation letter)	18	14.2
Total	127	100.0

**Table 3:** Ideal teacher characteristics for optimal cooperation.

Ideal Professor Characteristics for Optimal Cooperation	Ν	%
Patient, cooperative	28	22
Friendly, polite	26	20.5
Willing, accessible, available	25	19.7
Instructive	21	16.5
Other	27	21.3

## Data Availability

Data is contained within the article.

## Appendix A. The DSC Research Questionnaire

**DENTAL STUDENTS COOPERATION QUESTIONNAIRE (DSC)**

**A. Demographic Information**

**-Gender:**

Male……. Female…….

**-Parents educational level:**

Primary school……… Gymnasium………. Lyceum………. University……..

**-I have been involved in group work in the past:**

Yes……. No………

**-I would be interested in participating in teamwork again:**

Yes……... No………

**-Where am I going to practice dentistry?**

City center ……. Country region………. Island…….. Abroad………

I will not practice clinical dentistry………

**B. Investigation of Cooperation and Communication Attitudes**

Every question should be answered. Please read each statement and fill in the appropriate box according to the categorization of 1–5: 1 Strongly disagree, 2 Disagree, 3 Neutral, 4-Agree, 5 Strongly agree

	1. Strongly disagree	2. Disagree	3. Neutral	4. Agree	5. Strongly agree
The following questions investigate whether the students found the project interesting					
1. The cooperation and communication with my fellow students were interesting.					
2. The group project caught my interest.					
3. The project’s subject motivated me to work harder.					
4. I enjoy group projects.					
The following questions investigate whether the students found the project pleasant					
5. It was pleasant to listen to my fellow students’ ideas.					
6. The working environment was pleasant.					
7. There were differences between the group members.					
The following questions investigate whether the communication among the students was effective					
8. The group members cooperated efficiently.					
9. We managed to achieve group goals.					
10. All the members contributed equally.					
11. The group was supportive of all the members to achieve the project’s goals.					
12. The sense of responsibility encouraged me to work more efficiently.					
13. Taking initiatives helped in achieving group goals.					
14. The competition among the members caused conflicts, which negatively affected the goal of the project.					
The following questions investigate whether the communication among the students was helpful					
15. The group project helped me to have a better result than an individual project.					
16. The group project helped to make the most of each member.					
17. The project helped me realize my skills.					
18. The group project helped me understand my disadvantages.					
19. The group project helped me improve some of my disadvantages.					
20. The project helped to reduce the competition among us.					
21. The group project helped me realize the importance of communication in my future profession.					
The following questions investigate the effects of reward among students					
22. A reward at the end of the group project will motivate me to work more efficiently.					
23. A reward at the end of a group project would push me to participate in the project that I would not normally be interested in.					
24. I prefer a reward that concerns each person individually.					
25. Punishing people who do not cooperate would have a good impact on the team’s cooperation.					
26. I am rewarded by my parents for my work/grades.					
The following questions investigate the effects of motivation among students					
27. It would motivate me to work harder if we were examined more often and not just at the end of the work.					
28. The competition between the members pushed me to work harder.					
29. If the other members of the group were my friends, I would be motivated to work more efficiently.					
30. I could have a leadership-organizational role in the project.					
How modern technology helped the team cooperate due to lockdown					
31. We communicated through social media during lockdown to finish the project.					
32. Communication through social media helped to develop cooperation between the members due to lockdown.					
33. The group project helped me stay active during lockdown.					

**C. Investigation of Communication Skills**

	1 Strongly disagree	2 Disagree	3 Neutral	4 Agree	5 Strongly agree
Communication Skills					
1. Cooperation helped me in conflict management.					
2. Cooperation helped me put myself in the other person’s shoes.					
3. Cooperation helped me develop active listening skills.					
Thinking and Reasoning Skills					
4. Group project was more effective in developing critical thinking compared to lectures.					
5. Cooperation helped me in decision making.					
6. Cooperation helped me solve problems.					
7. Cooperation helped me develop trust with the other students.					
8. Cooperation helped me in my self-awareness.					
9. Cooperation helped me handle difficult people.					

**D. Choose One of the Following Answers:**

1. What reward would motivate me to work more efficiently?
  ○ Higher semester grade.
  ○ Certificate of participation in group projects.
  ○ Money reward.
  ○ Other (please explain)
2. How would I like my teacher in team working coaching? What characteristics would I like him to have in order to cooperate better with him?

(please explain)
